# Fabrication of Ultra-High Aspect Ratio (>420:1) Al_2_O_3_ Nanotube Arraysby Sidewall TransferMetal Assistant Chemical Etching

**DOI:** 10.3390/mi11040378

**Published:** 2020-04-03

**Authors:** Hailiang Li, Changqing Xie

**Affiliations:** Key Laboratory of Microelectronic Devices & Integrated Technology, Institute of Microelectronics of Chinese Academy of Sciences, Beijing 100029, China; lihailiang@ime.ac.cn

**Keywords:** Al_2_O_3_ nanotube, ultra-high aspect ratio, gold (Au) metal assisted chemical etching, atomic layer deposition, anisotropic dry etching

## Abstract

We report a robust, sidewall transfer metal assistant chemical etching scheme for fabricating Al_2_O_3_ nanotube arrays with an ultra-high aspect ratio. Electron beam lithography followed by low-temperature Au metal assisted chemical etching (MacEtch) is used to pattern high resolution, high aspect ratio, and vertical silicon nanostructures, used as a template. This template is subsequently transferred by an atomic layer deposition of the Al_2_O_3_ layer, followed by an annealing process, anisotropic dry etching of the Al_2_O_3_ layer, and a sacrificial silicon template. The process and characterization of the Al_2_O_3_ nanotube arrays are discussed in detail. Vertical Al_2_O_3_ nanotube arrays with line widths as small as 50 nm, heights of up to 21 μm, and aspect ratios up to 420:1 are fabricated on top of a silicon substrate. More importantly, such a sidewall transfer MacEtch approach is compatible with well-established silicon planar processes, and has the benefits of having a fully controllable linewidth and height, high reproducibility, and flexible design, making it attractive for a broad range of practical applications.

## 1. Introduction

In recent years, Al_2_O_3_ nanotube arrays possessing high surface-to-volume ratios have attracted much attention, owing to their potential applications in optoelectronics [1,2], biotechnology, and photocatalysis [3,4]. For example, several recent studies have indicated that Al_2_O_3_ nanotube arrays exhibit excellent dielectric properties [5] and good flexibility [6], as compared with other oxide nanotubes. Thus, Al_2_O_3_ nanotube arrays would be more advantageous for use as optical transportation media in optoelectronics. They can also be utilized as a new biomineralization nanoreactor in biotechnology, and so on. Various template-based strategies for the fabrication of Al_2_O_3_ nanotubes with a high aspect ratio have been proposed. These include hydrothermal reaction methods [7], coating of carbon nanotubes with aluminum isopropoxide [8], anodization of Si-based Al film [9,10], and etching of a porous anodic alumina (PAA) template in a NaOH solution and ultrasonic treatment of PAA membrane [11,12]. In addition to the Al_2_O_3_ nanotubes, atomic layer deposition (ALD) coatings of silicon oxide (SiO_2_) [13], zinc oxide (ZnO) [14,15], zirconium dioxide(ZrO_2_) [16], and titanium oxide(TiO_2_) [16,17] have also been successfully applied to the preparation of nanostructures.Using these methods, much progress has been made on achieving highly ordered nanotube arrays with a controllable size and short length. However, due to the poor consistency of the pillar size in the templates, it is not easy to confine and control the profile of the fabricated nanostructures. Moreover, separating hollow nanostructures from a template to obtain individual nanostructures remains a challenge. Many kinds of nanotubes with high aspect ratios will lose their tubular structure after extraction from the template. With the continuous request for high performance nanotube-based devices, the aspect ratio of the fabricated nanotube arrays as obtained by tailoring electrochemical conditions is far from satisfactory.

Metal assisted chemical etching (MacEtch), first reported by Li and Bohn in 2001 [18], offers a promising wet etching solution for generating silicon nanostructures [19,20,21,22]. In addition, the MacEtch of germanium (Ge) [23] and III−V compound semiconductors, such as GaN [24,25], GaAs [26,27,28], GaP [29], InP [30], AlGaAs [31], InGaAs [32], and InGaP [27], have also been demonstrated. This method has the benefits of inherent simplicity, a low cost, high versatility, and high reproducibility, making it attractive for preparing silicon nanowire arrays. The conventional method for generating micrometer, submicrometer, and nanosized silicon structures with high aspect ratios [19,20,21,22] is by submerging a metal-coated Si sample into an etchant solution composed of hydrofluoric acid, hydrogen peroxide solution, and a diluting agent. Furthermore, large-area uniform micro-gratings with well controlled morphological features and depths as large as 80 μm have also been successfully produced by optimizing the MacEtch method [33]. Highly dense Si/TiO_2_ core/shell nanowire arrays have also been synthesized using a nanostructured Si template obtained by the MacEtch of the Si substrates and a layer of TiO_2_ deposited by ALD [34,35]. In the previous works, using Ti/Au nanostructures patterned with electron-beam lithography followed by ion beam etching, we fabricated vertical silicon nanopillar arrays with a period of 250 nm and an aspect ratio of 160:1 using MacEtch [36]. A 20 nm minimum feature size was also realized by MacEtch-based nanoimprinting [37]. Unfortunately, the MacEtch method cannot be directly adopted to generate oxide nanotubes. 

Sidewall transfer lithography has been widely recognized as a promising technology that can fabricate deca-nanometer metal-oxide-semiconductor field-effect transistors (MOSFETs) and siliconnanostructures [38,39,40]. The key idea is to combine three well-established techniques (lithography, sidewall process, and dry etching) to create silicon nanostructures. In this work, we present a reliable means, called sidewall transfer metal assistant chemical etching, to fabricate Al_2_O_3_ nanotube arrays with an ultra-high aspect ratio. The key idea is to combine a low-temperature MacEtch and sidewall transfer process based on ALD and dry etching to simultaneously achieve their respective advantages. The former is used to generate vertically oriented silicon nanostructures that serve as a sacrificial layer, while the latteris used to transfer the silicon nanostructures to Al_2_O_3_ nanotube arrays with a higher aspect ratio and smaller feature size. The ultimate aspect ratio and feature size of the fabricated Al_2_O_3_ nanotube arrays can be controlled by modifying the thickness of the Al_2_O_3_ film deposited by ALD. The influence of the deposition temperature and annealing temperature on the structure and optical properties of Al_2_O_3_ thin films deposited by ALD are also examined.

## 2. Materials and Methods 

**Figure 1:**
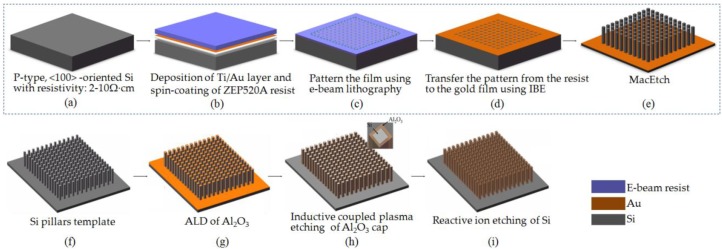
Schematic diagram of the sidewall transfer MacEtch process for the realization of the Al_2_O_3_ nanotube arrays with ultra-high aspect ratios: (**a**) silicon substrate; (**b**) deposition of the Ti/Au layer and ZEP520A resist; (**c**) electron beam lithography; (**d**) ion beam etching; (**e**) metal assisted chemical etching (MacEtch); (**f**) Si pillars template; (**g**) atomic layer deposition (ALD) Al_2_O_3_ film; (**h**) dry etching; (**i**) reactive ion etching of Si.

The process flow of the proposed sidewall transfer MacEtchis shown in Figure 1. In our experiments, 4in.p-Type<100>CZsiliconwafers (Silicon Quest International, San Jose, CA, USA) with a resistivity of 2–10 Ω·cm and thickness of 500 µm were used. After the samples (Figure 1a) were cleaned in sulfuric acid and hydrogen peroxide to remove the surface native oxide layers, a 3-nmTi/20 nm Au thin film was deposited on the silicon substrates by a magnetron sputtering system (ACS-4000, ULVAC Company, Chigasaki, Japan). The working pressure was maintained at 4.5 × 10^−6^ Torr, and the temperature of the chamber was kept at 25 °C over the entire deposition process. Then, highly sensitive chlorinated electron beam resist ZEP520A was spin-coated on the Ti/Au layer to a thickness of about 400 nm, and was bakedon a hotplate at 180 °C for 2 min (Figure 1b). The resist was exposed with an electron beam lithography system (JBX-6300FS, JEOL Company, Tokyo, Japan) for patterning definition (Figure 1c), and ion beam etching (IBE) was performed to transfer ZEP520A resist pattern onto the Ti/Au layer, forming Ti/Au nanostructures that serve as a local cathode (Figure 1d). Then, the MacEtch processwas carried out to generate silicon nanostructures with a high aspect ratio (Figure 1e). The ALD process was used to deposit the Al_2_O_3_ film on the sidewalls and on top of the generated silicon nanostructures (Figure 1g). Finally, the exposed tops of the Al_2_O_3_ film and its silicon nanostructures underneath were removed by dry etching, forming Al_2_O_3_ nanotube arrays with a higher aspect ratio and smaller feature size.

The morphology and structure of the fabricated Al_2_O_3_ nanotube arrays were characterized by a scanning electron microscope (SEM; SUPRA-55, Zeiss, Oberkochen, Germany). The information on the surface chemistry of the Al_2_O_3_ layer deposited by the atomic layer deposition (ALD) technique was investigated using X-ray photo emission spectroscopy (XPS) with a monochromatic Al Kα X-ray source. Both the Al_2_O_3_ film thickness and its refractive index profile were determined by a spectroscopic ellipsometry (Horiba Uvisel FUV, Kyoto, Japan) over the spectral range of 150 to 900 nm using the Cauchy model.

### 2.1. Fabrication ofTi/Au Nanostructures with Low Aspect Ratio

To obtain high-resolution resist patterns, electron beam lithography was performed at an accelerating voltage of 100 kV, with a beam current of 200 pA and an exposure dose of 400 µC/cm^2^. After electron beam exposure, the ZEP520A resist was developed using a standard developer N50D (ZEON) for 1 min at 18 °C, and rinsed with isopropanol (IPA) for 40 s to stop development. Then, the sample was dried with a steady stream of N_2_.

Next, a home-madeargon (Ar) IBE system was used to transfer the resist patterns into a Ti/Au thin film deposited onto the silicon wafer. The working pressure was maintained at 1.0 × 10^−4^ Torr, and the substrate temperature was lower than 100 °C over the entire IBE process. The Ar^+^ ion energy and the beam current density were 500 eV and 1 mA/cm^2^, respectively. The corresponding etching rates of Au and the resist were 120 nm/min and 20 nm/min, respectively, resulting in a selectivity ratio of 6:1. The etching time in our experiments was 12 s. After completion of the IBE process, the residual ZEP520A resist was removed by washing with a resist removal solution (ZDMAC, ZEON Company, Tokyo, Japan), followed by oxygen plasma ashing. The patterned Ti/Au nanostructures served as a catalyst in the subsequent low-temperature MacEtch process.

### 2.2. Fabrication of Silicon Nanostructures with High Aspect Ratio

A low-temperature Au MacEtch process was performed to generate silicon nanostructures with a high aspect ratio. It should be noted that samples must be kept clean and tidy before the Au MacEtch process. The Au MacEtch process was carried out in an etchant solution composed of hydrofluoric acid, hydrogen peroxide solution, and deionized water (4.1 M HF/0.15 M H_2_O_2_/45 M H_2_O) at 2 °C. The etching was conducted for 15 min. It is well known that the collapse of large aspect ratio nanostructures often occurs during the drying step. In our drying scheme, we used isopropanol with low surface tension, instead of deionized water (DI) water as a rinse solution. This reduced the capillary force acting on the silicon nanostructures. After the sample was rinsed with isopropanol, it was evaporated naturally and dried at room temperature.

### 2.3. Al_2_O_3_ Film Deposition by ALD

The ALD process allows one to precisely deposit highly uniform and conformal thin films onto complex three-dimensional topographies. Here, an Al_2_O_3_ film was deposited using Al(CH_3_)_3_ (TMA) and deionized water with a hot-wall atomic layer deposition system (Picosun R200, Espoo, Finland). The deposition temperature was 300 °C. An Al_2_O_3_ film was grown on the sidewalls and on top of the fabricated silicon nanostructures with an aspect ratio of 160:1, using TMA and deionized water. TMA (Sigma Aldrich, St. Louis, MO, USA) was used as the precursor and deionized water was used as an oxidant source during the ALD process. The TMA reactant exposure time, N_2_ purge time following TMA reactant exposure, water exposure time, and N_2_ purge time following the H_2_O reactant exposure were 0.5 s, 2 s, 0.5 s, and 2 s, respectively. The growth rate of the Al_2_O_3_ film was 0.089 nm/cycle, as inferred by spectroscopic ellipsometry. The sample was processed with 400 ALD cycles, and the corresponding Al_2_O_3_ film thickness was 50 nm.

### 2.4. Annealing Processand Characterization of the Al_2_O_3_ Film

The thermal expansion coefficient of Al_2_O_3_ film is 8.2 × 10^−6^ °C^−1^, which is higher than that of silicon (2.6 × 10^−6^ °C^−1^). To compensate the lattice mismatch between the Al_2_O_3_ ALD filmand silicon nanostructures, four samples were annealed at 700 °C, 800 °C, 900 °C, and 1000 °C under a vacuum for 90 min, seperately. The specifications of the Al_2_O_3_ film was investigated using X-ray photoelectron spectra (XPS). Characterization of the optical properties of our Al_2_O_3_ film from 150 to 900 nm wavelengths was also performed using spectroscopic ellipsometry. 

### 2.5. Formation of Al_2_O_3_ Nanotube Arrays with an Ultra-High Aspect Ratio

First, the exposed tops of the Al_2_O_3_ film on the silicon nanostructures were removed by an inductive coupled plasma (ICP) etching system (ULVAC, Japan), with a mixture of BCl_3_ and Cl_2_ reactive gas. Secondly, the silicon nanostructures underneath the exposed tops of the Al_2_O_3_ film were removed by the same ICP etching system with SF_6_ reactive gas and an isotropic reactive ion etching mode. The etching parameters of the Al_2_O_3_ film and silicon nanostructuresa re summarized in Table 1.

The etch rates of the Al_2_O_3_ film and silicon nanostructures were 0.89 nm/s and 9 nm/s, respectively. The etching selectivity between the silicon nanostructures and Al_2_O_3_ film was as high as 66,000:1 for the Si etching recipe [41].

## 3. Results and Discussion

**Figure 2:**
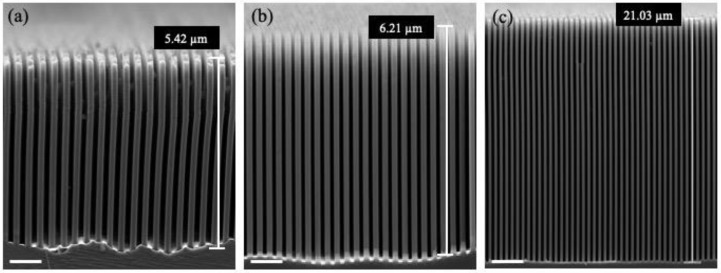
Cross-sectional scanning electron microscope (SEM) micrographs of silicon nanostructures using various etchant solutions composed of hydrofluoric acid, hydrogen peroxide solution, and deionised (DI) water with molar ratios of (**a**) 4.8:0.35:50, (**b**) 4.8:0.15:50, and (**c**) 4.8:0.15:50. The scale bars in (**a**–**c**) are 850 nm, 900 nm, and 1.9 µm, respectively. The etching temperatures in (**a**,**b**) are 22 °C, and the etching temperature in (**c**) is 2 °C.

Figure 2 shows the dependence of the Au MacEtch process parameters (etchant solutions and etching temperature) on the quality of the fabricated silicon nanostructures. When the hydrogen peroxide concentration in the etchant solution was relatively high, the lateral etching rate increased and defects began to occur on the sidewalls, as shown in Figure 2a. This is because as the concentration of H_2_O_2_ increased, the number of the generated holes also increased, resulting in an increase in the silicon etching rate. In other words, when the generated holes between the Si and Ti/Au interfaces could not be completely consumed, the excess holes spread laterally, leading to lateral corrosion and the formation of defects in the sidewalls. Using an optimized H_2_O_2_ concentration, the transverse etching rate could be limited by the availability of the generated hole. Thus, vertical silicon nanostructures could be obtained, as shown in Figure 2b.

Etching temperature also plays an essential role in the Au MacEtch process. When the Au MacEtch process was performed at 2 °C, a better morphology could be obtained than that at room temperature, at the expense of a much lower etching rate. In this work, a low-temperature Au MacEtch process could result in silicon nanostructures with an aspect ratio of up to 160:1, as shown in Figure 2c. Silicon nanostructure bottoms that are especially clean and flat can be obtained.

**Figure 3:**
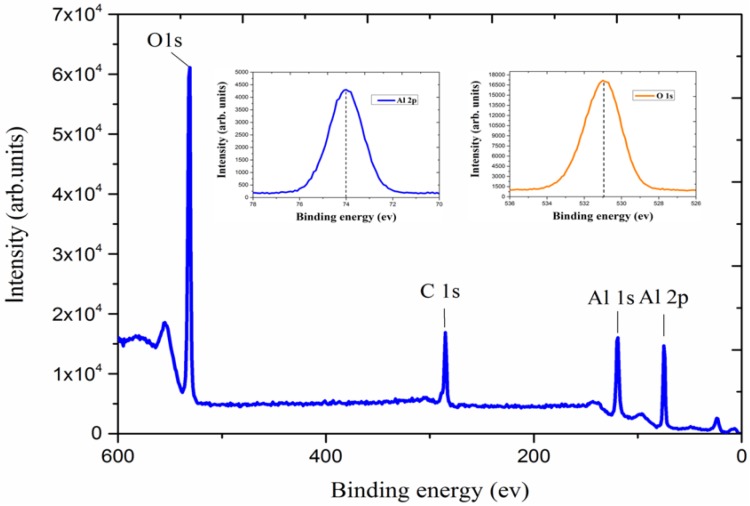
X-ray photoelectron spectra of the Al_2_O_3_ layer deposited at 300 °C. The two insets show the Al (2p) and O (1s) peaks, respectively.

Figure 3 shows the binding energies of the Al_2_O_3_ film deposited at 300 °C. Two signatures of orbital, 74.4 eV for Al (2p) and 531.5 eV for O (1s), can be observed from an XPS wide scan. The O/Al ratio of the Al_2_O_3_ film is close to the expected value of 1.5, corresponding to the lattice oxygen of Al_2_O_3_ [42]. The difference between these two elemental peaksis close to the standard values in the literature for different forms of aluminum oxide [43,44]. A peak of binding energy of 1s carbon in the Al_2_O_3_ film could also be observed, indicating either an incomplete reaction or an insufficient N_2_ purge time.

**Figure 4:**
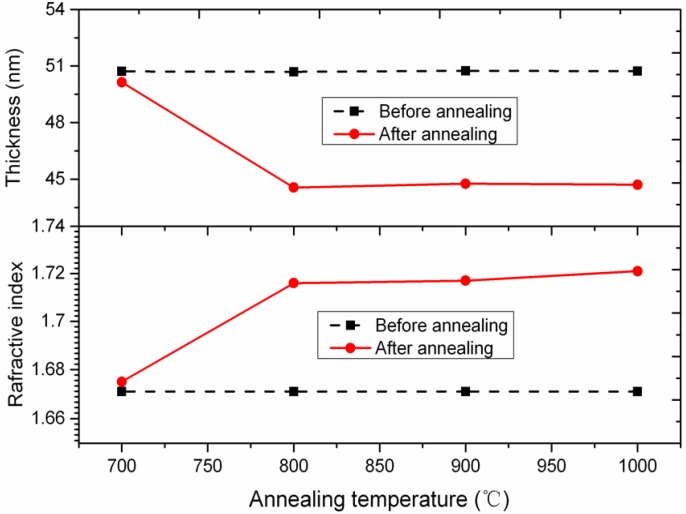
Variation of film thickness and refractive index versus annealing temperature.

Figure 4 shows the Al_2_O_3_ ALD film thickness and refractive index as a function of the annealing temperature. The refractive index of the Al_2_O_3_ ALD filminitially increased with an increase of annealing temperature from 700 °C to 800 °C, and later saturated with the increasing annealing temperature. When the annealing temperature reached 1150 °C, the refractive index approached a maximized value of 1.724, which is slightly smaller than that of crystalline sapphire (1.76). The measured data indicate that the amorphous Al_2_O_3_ ALD film in this experiment would densify further upon crystallization. This is consistent with the existing result [45]. Meanwhile, there was a 10% decrease in the Al_2_O_3_ ALD film thickness with increasing the annealing temperature from 700 °C to 800 °C, which was mainly due to an increase inthe density and purity levels of the Al_2_O_3_ films deposited by ALD. The measured data also indicate the occurrence of Al_2_O_3_ crystallization after high-temperature annealing, which appears to be particularly advantageous for preventing the collapse of ultra-high aspect ratio Al_2_O_3_ nanotube arrays.

**Figure 5:**
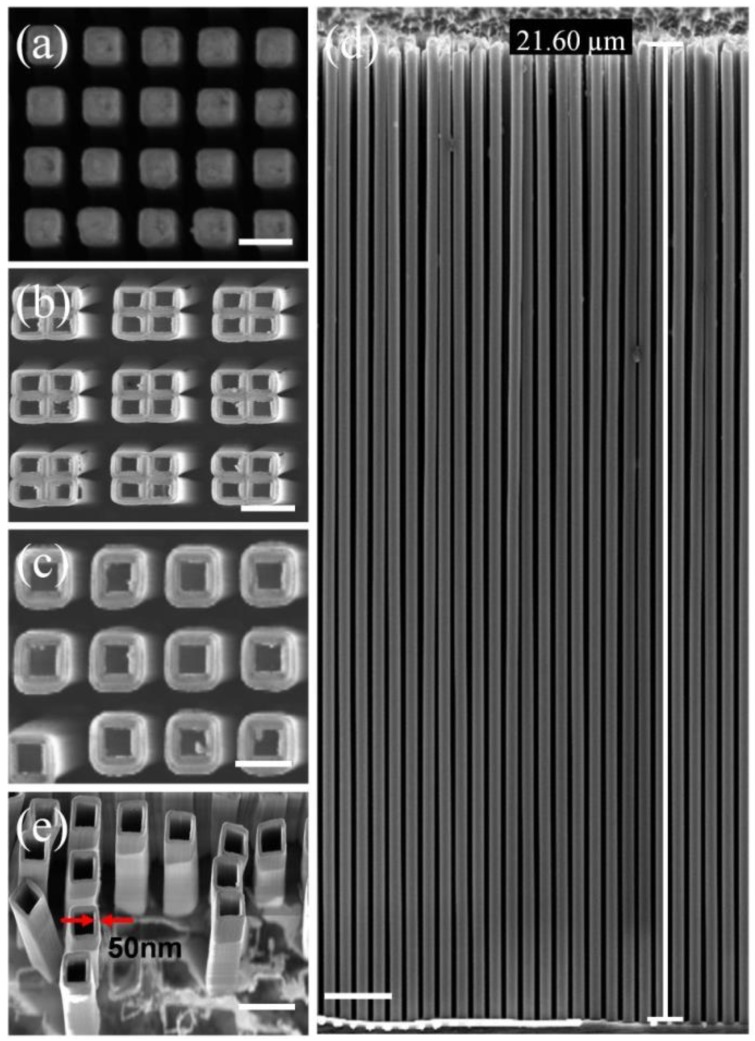
A series of images demonstrating sidewall transfer MacEtch for the preparation of ultra-high aspect ratio Al_2_O_3_ nanotube arrays. (**a**) Top view SEM of the Si nanostructures after Al_2_O_3_ film deposition by ALD. The scale bar is 300 nm. (**b**) Top view SEM of the generated Al_2_O_3_ nanotube arrays without annealing process. The scale bar is 400 nm. (**c**,**d**) SEM images of the top and cross section views of the generated Al_2_O_3_ nanotube arrays resulting from the sidewall transfer MacEtch process. The scale barsin (**c**,**d**) are 250 nm and 1.6 µm, respectively. (**e**) Tilted SEM view of Al_2_O_3_ nano-tube structures after the whole process. The scale bar is 400 nm. The tilting angle is 35°.

Figure 5a shows the SEM image of the top view of the generated Si nanostructures, followed by the Al_2_O_3_ film deposition by ALD and annealedat 1150 °C for 90 min. The results of the generated Al_2_O_3_ nanotube arrays without the annealing process are also given in Figure 5b. One can clearly see that the Al_2_O_3_ nanotube arrays bend and lean against each other.The bending is probably caused by a mismatch in internal stress between the Al_2_O_3_ layer and Si pillar. The thermal expansion coefficient of the Al_2_O_3_ film is 8.2 × 10^−6^ °C^−1^, while the thermal expansion coefficient of silicon is 2.6 × 10^−6^ °C^−1^. It should be noted that the Al_2_O_3_ ALD film was deposited by the temperature gradient method over a temperature range from room temperature to 300 °C, and the Si cores were removed afterwards using a plasma reactive ion etching at 25 °C. By comparison, Figure 5c,d shows the SEM images of the top andcross section views, respectively, of the generated Al_2_O_3_ nanotube arrays after the whole process was finished. These uniform structures had a line width of 50 nm and height of 21 μm, corresponding to an aspect ratio as high as 420:1. The Al_2_O_3_ nanotube arrays were perfectly preserved from collapsing after the annealing process, confirming the successful pattern transfer of the sidewall transfer MacEtch process. The shape of the Al_2_O_3_ nanotube arrays exhibited very little deformation relative to that of the original Si nanostructures. Figure 5e shows a tilted SEM view of the Al_2_O_3_ nanotube arrays after the whole process. One can see that the silicon template was almost completely removed by plasma reactive ion etching at 25 °C.

## 4. Conclusions

In summary, we have demonstrated a reliable sidewall transfer MacEtch process to fabricate ultra-high aspect ratio Al_2_O_3_ nanotube arrays with linewidthsas small as 50 nm, heights of up to 21 μm, and an aspect ratio of up to 420:1. This technique combines the advantages of the high aspect ratio nanostructure capabilities of the low-temperature Au MacEtch with thesidewall transfer process. The use of the sidewall transfer process has two advantages. First, it leads to higher-resolution and higher aspect ratio Al_2_O_3_ nanotube patterns than with MacEtch, and second, the line-width of the Al_2_O_3_ nanotubes can be precisely controlled by the cycle number of ALD for Al_2_O_3_. The sidewall transfer MacEtch provides a promising route to the scalable manufacturing of ultra-high aspect ratio Al_2_O_3_ nanotube arrays, and is applicable to other kinds of oxide nanotubes, as long as the oxide can be deposited by ALD.

## Figures and Tables

**Table 1:** Recipes for Al_2_O_3_ and Si in etch system.

Process Parameters	Al_2_O_3_ Etch	Si Etch
Cl_2_ (sccm)	1.2	−
BCl_3_ (sccm)	6.8	−
SF_6_ (sccm)	−	90
Pressure (mtorr)	3	4
Coil power (W)	900	400
Platen power (W)	200	3

Process temperature is 25 °C for all of the processes.
